# Implementation and impact of simulated patient perspective exercises in undergraduate nursing education: A scoping review

**DOI:** 10.3205/zma001847

**Published:** 2026-04-15

**Authors:** Anna Christine Steinacker, Michael Klingenberg, Stefan Bösner

**Affiliations:** 1 University of Applied Sciences Fulda, Fulda, Germany; 2 Philipps University Marburg, Marburg, Germany

**Keywords:** education nursing, simulation training, empathy, experiential learning, embodied learning

## Abstract

**Objectives:**

Exercises that immerse nursing students in the patient perspective are increasingly used to support empathy and reflective learning. This scoping review aimed to map the current literature on these exercises in undergraduate nursing education, focusing on their design, reported effects, and pedagogical implications.

**Methods:**

Following Arksey and O’Malley’s framework and PRISMA-ScR guidelines, we searched PubMed, CINAHL, Embase, and Web of Science, completing the final database search in Nov 2024. Eligible studies included peer-reviewed empirical research involving undergraduate nursing students. Relevant data were extracted, categorized using a standardized charting form, and synthesized thematically to identify common patterns across studies.

**Results:**

Twenty-two studies published between 2009 and 2024 were included in this scoping review. Studies used qualitative, quantitative or mixed-method designs and examined simulations focused on aging, disability, or mental health. Three key themes were identified: (1) students’ responses during the simulation exercises, (2) perceived impact after the exercises and (3) ethical and pedagogical considerations related to implementation.

**Conclusions:**

Patient perspective simulations may enhance empathy and support reflective practice among nursing students. However, ethical concerns, questions of authenticity, and varied implementation approaches highlight the need for thoughtful design. Further research should explore long-term effects and establish best practices for integrating these exercises into nursing curricula.

## Zusammenfassung

**Zielsetzung:**

Selbsterfahrungsübungen zur Förderung der Perspektivübernahme aus Sicht von Patient:innen gewinnen in der pflegerischen Hochschulausbildung zunehmend an Bedeutung. Ziel dieses Scoping Reviews ist es, die aktuelle Literatur zu solchen Übungen systematisch zu erfassen und hinsichtlich Gestaltung, berichteter Effekte und pädagogischer Implikationen zu strukturieren.

**Methoden:**

Basierend auf dem Rahmen von Arksey und O’Malley sowie den PRISMA-ScR-Leitlinien wurde eine systematische Literaturrecherche in PubMed, CINAHL, Embase und Web of Science durchgeführt (letzte Suche: November 2024). Eingeschlossen wurden peer-reviewte empirische Studien mit Pflegestudierenden. Die Daten wurden standardisiert extrahiert und thematisch synthetisiert.

**Ergebnisse:**

Insgesamt wurden 22 Studien (2009-2024) eingeschlossen, die qualitative, quantitative oder Mixed-Methods-Designs verwendeten. Die Selbsterfahrungsübungen fokussierten überwiegend auf Alter, Behinderung oder psychische Gesundheit. Drei Themenbereiche wurden identifiziert: (1) unmittelbare Reaktionen der Studierenden, (2) wahrgenommene Effekte nach den Übungen und (3) ethische sowie pädagogische Aspekte.

**Schlussfolgerungen:**

Selbsterfahrungsübungen können Empathie und reflexive Praxis bei Pflegestudierenden fördern. Gleichzeitig verdeutlichen ethische Fragestellungen und heterogene Umsetzungsformen die Notwendigkeit einer sorgfältigen didaktischen Gestaltung. Zukünftige Forschung sollte Langzeiteffekte untersuchen und Best-Practice-Ansätze für die curriculare Integration entwickeln.

## 1. Introduction

In nursing education, as in other health-related fields, patient perspective exercises are increasingly used to immerse students in the care experience from the viewpoint of those receiving it [1], [2]. Also known as “disability simulations” or “point-of-view simulations,” these activities aim to facilitate a shift in perspective by helping students better understand the physical and emotional challenges associated with illness or disability, such as using a wheelchair, living with visual or auditory impairments, or requiring assistance with daily activities [3], [4]. This pedagogical approach is particularly relevant in nursing education, where students engage in intimate and sustained patient interactions that go beyond technical competence. Nursing is a profession in which the body plays a central role, both the patient's and the nurse’s. As Oelke [5] notes, recognizing one’s own bodily and emotional responses is essential for compassionate care. Educators thus play a key role in fostering this awareness through experiential methods that promote empathy, reflection, and clinical reasoning [6].

Despite their popularity, perspective-taking exercises raise critical questions about educational effectiveness and ethical implications. A recurring critique is that these simulations tend to emphasize the initial “shock” of acquiring a disability, such as sudden blindness or mobility loss, while neglecting the longer-term processes of adaptation, identity reconstruction, and resilience [4], [7], [8]. Such portrayals risk oversimplifying complex realities and may unintentionally reinforce stereotypes or provoke pity rather than authentic empathy [1], [9], [10]. These simulations often focus on technical tasks, like maneuvering a wheelchair, without fully capturing the emotional, social, or systemic dimensions of illness or disability. Students may experience strong emotional responses, including fear or discomfort, and some report difficulties separating their own imagined suffering from patients’ lived realities [1], [11], [12].

Nevertheless, many educators emphasize the teaching value of these exercises. When paired with thoughtful prebriefing and debriefing, simulations can support the development of empathy, help students better grasp the patient perspective, and contribute to their professional growth [2], [4], [13]. At the same time, outcomes are not always consistent, and there is increasing attention to the ethical dynamics between students and educators, as well as the need for greater inclusion of individuals with lived experiences in designing such interventions [1], [8], [9], [11]. 

While some studies report positive outcomes, others show inconsistent or short-lived effects. Implementation practices likewise vary considerably, and there is currently no consensus on best practices or guiding pedagogical frameworks [13]. These concerns are gaining traction across health professions education, but little research has focused specifically on undergraduate nursing programs.

To address this gap, the present scoping review maps the existing literature on simulated patient perspective exercises in undergraduate nursing education. The review aims to

1. identify the types and designs of exercises employed,
2. examine students’ experiences and reported outcomes, and
3. explore the ethical, practical, and pedagogical implications of implementing such exercises in nursing curricula.

Given the breadth of this topic and the need for a comprehensive overview, a scoping review methodology was chosen to clarify conceptual boundaries and identify areas for future research [14].

To guide this review, the following research question and sub-questions were explored:

*What types of patient perspective exercises are employed in undergraduate nursing education, and how are these exercises experienced and evaluated by nursing students?*

1. What emotional and physiological responses do students report during participation in these exercises?
2. What challenges, limitations, or opportunities do educators encounter when implementing patient perspective exercises?

## 2. Methods

### 2.1. Scoping review design

This scoping review was conducted following the methodological framework of Arksey and O’Malley [14], which outlines five stages: identifying the research question, identifying relevant studies, study selection, charting the data, and collating, summarizing, and reporting results. In line with evolving scoping review methodology, recommendations by Peters et al. [15] were also incorporated, particularly concerning iterative data charting and transparent reporting. The review is reported in accordance with the Preferred Reporting Items for Systematic Reviews and Meta-Analyses extension for Scoping Reviews (PRISMA-ScR) [16]. The review protocol is available from the authors upon request.

### 2.2. Information sources and search strategy

We conducted a comprehensive literature search in four databases: PubMed, CINAHL, Embase, and Web of Science. The final database search was executed in November 2024. The search strategy was structured around three key concepts:

- Concept 1: Nursing education (e.g., undergraduate nursing education, pre-licensure nursing training)
- Concept 2: Patient perspective exercise (e.g., disability simulation, perspective training, point-of-view simulation)
- Concept 3: Keywords related to outcomes (e.g., empathy, compassion, attitudes, understanding, care)

The concept and keywords used to develop the search strategy in PubMed are provided in table 1 (Tab. 1), while the exact PubMed search strategy, as executed, is included in attachment 1 , along with the number of records retrieved per line/concept. Full strategies for the other databases can be requested from the authors.

Forward and backward citation tracking of all included studies was also performed. All references were imported into Citavi reference management software, where automatic and manual deduplication was performed.

### 2.3. Selection of sources of evidence

To be included in this review, studies had to be empirical and peer-reviewed, published between 2009 and 2024 to capture recent results, and focus on undergraduate nursing students. Studies that included participants from other disciplines were eligible if nursing students formed a part of the sample. Eligible studies described embodied patient perspective exercises designed to simulate illness, disability, or aging from the viewpoint of the patient. Additionally, studies were required to report on relevant outcomes, including empathy, attitudes, emotional or physiological reactions, understanding, or learning outcomes related to the exercise.

We excluded literature reviews, theoretical papers, editorials, and conference abstracts, as well as studies published in languages other than English or German, due to language limitations within the research team. Furthermore, grey literature such as dissertations or unpublished reports was excluded to ensure that the review focused on peer-reviewed academic sources.

After deduplication, titles and abstracts were screened against eligibility criteria. Full texts were then assessed for inclusion. Screening was conducted by one author (the first author) due to resource constraints. To ensure methodological transparency and consistency, screening decisions were guided by predefined eligibility criteria and regularly discussed within the research team. Ambiguous cases were reviewed collaboratively until consensus was reached.

### 2.4. Data charting

A standardized charting form was used to capture study characteristics (author, year, country), type of simulation and implementation, student responses, reported outcomes (e.g., empathy, attitudes, emotional/learning outcomes), and pedagogical strategies (e.g., prebriefing, debriefing). Charting was conducted by the first author using MAXQDA software. To enhance rigor, two student assistants supported a second round of coding and data verification. We note that “student responses” (e.g., reflective writing, narratives) and “reported outcomes” (e.g., standardized measures, evaluations) represent distinct sources of data and were therefore analyzed separately.

Themes were developed following Braun and Clarke’s [17] approach to thematic analysis. This analytic framework was applied iteratively, supported by team discussions and interpretive synthesis in line with scoping review methodology. The results were narratively summarized, with tables used to present key study features and outcomes.

## 3. Results

### 3.1. Study selection

**Figure 1:**
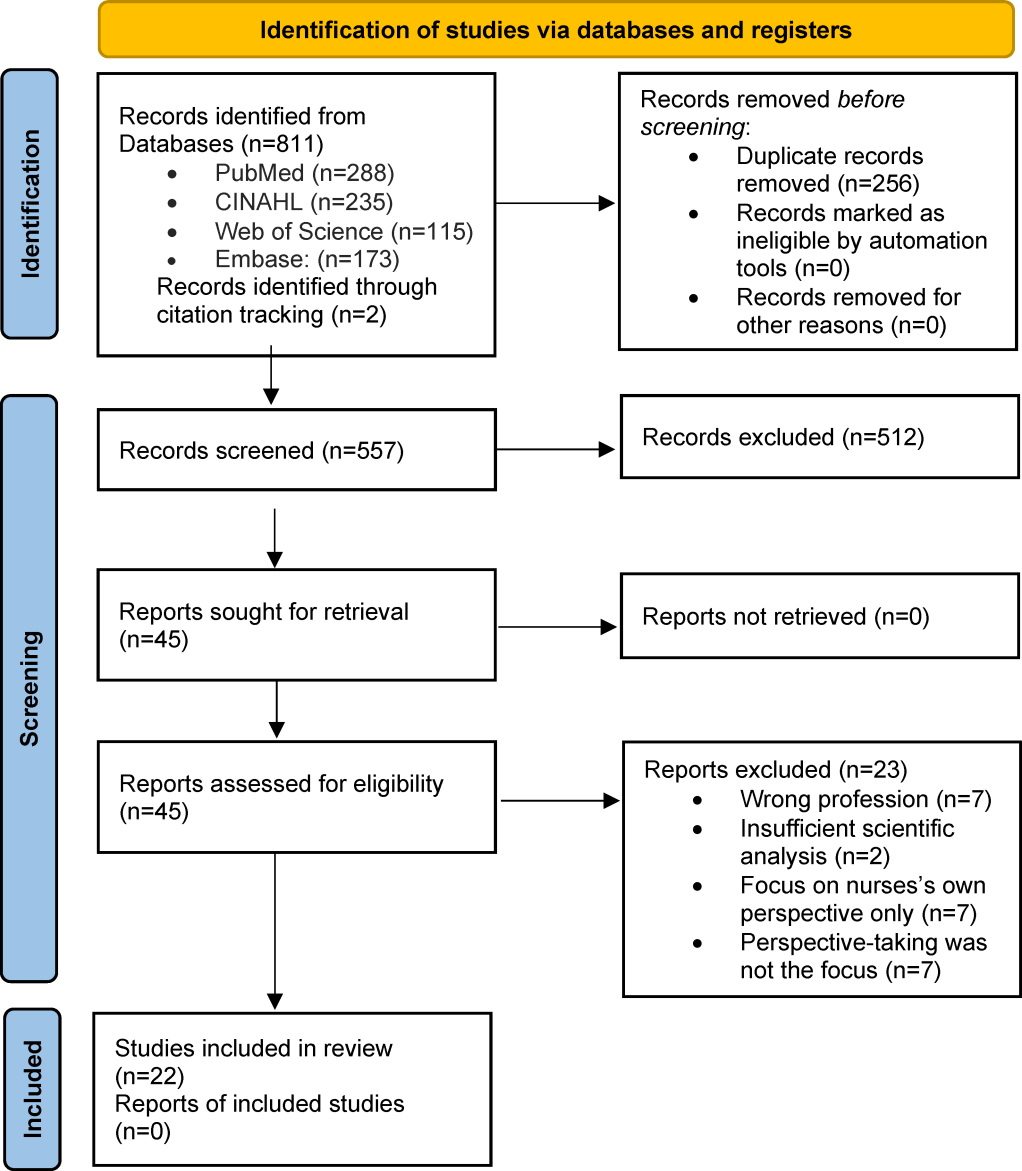
PRISMA Flow Chart [18]

A total of 22 studies were included in this review. The selection process is illustrated as a PRISMA flow diagram in figure 1 (Fig. 1) [18]. A total of 811 records were identified across the four databases and another 2 sources were identified through citation tracking. After deduplication, titles and abstracts were screened against eligibility criteria. Full texts were then assessed for inclusion. 

### 3.2. Characteristics of included studies

The included studies covered different simulation approaches aimed at improving empathy and understanding of patient experiences (see attachment 2 ). Methodologically, eleven studies employed qualitative, eight quantitative, and three mixed methods designs. Most studies were conducted in the United States (n=12), with others from Australia (n=4), Sweden (n=2), and one each from Türkiye, the Netherlands, Iran, and Taiwan.

### 3.3. Types and implementation of patient perspective exercises

The 22 included studies employed a variety of simulation formats to introduce perspective-taking and empathy among undergraduate nursing students. The most frequently used was a simulation exercise called “voices that are distressing” (n=6), in which participants listened to audio recordings simulating auditory hallucinations [19]. This format aimed to help students understand the psychological impact of mental health conditions such as schizophrenia [20], [21], [22], [23], [24], [25]. A “day in the life of a patient” approach was used in five studies, often combining multiple sensory or mobility limitations to reflect the complexity of living with a chronic condition [26], [27], [28], [29], [30]. Simulations focusing on wearing an ostomy appliance were conducted in four studies, aimed at increasing awareness of the emotional and practical difficulties faced by patients managing an ostomy [31], [32], [33], [34]. Two studies explored visual or hearing impairment simulations, one of which incorporated virtual reality (VR) elements to add immersion and the feeling of “being there” [35], [36]. Age simulation suits were used in two studies conducted by the same research team, one qualitative and one quantitative, to explore different aspects of aging-related challenges [37], [38]. Two studies used a program called “virtual dementia tour” intended to simulate the disorientation, frustration, and confusion associated with dementia [39], [40], [41]. One study looked at the process of HIV testing, allowing students to reflect on how patients may feel when going through sensitive medical diagnostics [42]. 

Implementation practices varied across studies, particularly in how students were prepared for and debriefed after the simulation exercises. Pre-briefing commonly included structured activities such as video introductions, theoretical framing, or scenario descriptions to help students orient themselves to the patient perspective [23], [29], [36], [40]. In some cases, pre-briefing also addressed emotional safety and provided guidance for students with lived experience [23]. Debriefing practices ranged from one-on-one facilitated sessions [41] to group discussions or written reflections aimed at consolidating learning and fostering empathy [20], [29], [31]. While the structure and content of these sessions varied, they were consistently used to help students process their emotional responses and connect the simulated experiences to real-world caregiving challenges. Some studies emphasized the importance of simulation realism, enhanced through the use of professional actors, VR tools, or collaboration with people who had lived experience with the condition being simulated [27], [40], [41]. In three studies, students rotated through different roles, e.g. taking the role of patients and nurses [21], [26], [27]. 

### 3.4. Themes

#### 3.4.1. Theme 1: Students’ responses during the simulation

Students frequently reported feelings of discomfort, frustration, fear, and heightened vulnerability while participating in the simulation exercises. The emotional responses reported by students ranged from anxiety, embarrassment, and insecurity to more intense responses such as panic, sadness, and a sense of being overwhelmed [22], [23], [31], [33], [34], [37]. Participants often described struggling to stay focused, complete basic tasks, or interact socially due to the sensory or cognitive challenges imposed during the simulations [20], [21], [35]. Students’ emotional and physiological responses varied depending on the type of simulation. For instance, exercises simulating auditory hallucinations were associated with distress, irritability, and a sense of being mentally overpowered [23]. Also, simulations involving visual impairment or mobility restrictions often triggered confusion, dependence, loneliness, and fear of physical harm [29], [35]. Students also expressed embarrassment related to the visibility of assistive devices, such as ostomy bags, and reported self-consciousness and concerns about social stigma [31], [33], [34]. Students even reported feeling physical symptoms such as tachycardia and discomfort [42], and some described feeling “trapped in their own bubble”, unable to connect with those around them [38]. 

#### 3.4.2. Theme 2: Perceived impact after the simulation exercises 

Despite the challenges encountered during the simulations, many students expressed gratitude for having participated, describing the experiences as powerful, eye-opening, and transformative [20], [21], [23], [38], [41]. Students repeatedly stated that the simulations offered a kind of insight that could not be achieved through textbooks or clinical placements alone, with one participant emphasizing that “personal experience stays with you” [23]. The authors reported that the simulations fostered deeper awareness and empathy by allowing students to momentarily “step into the shoes” of individuals living with mental illness, sensory impairments, or age-related decline [22], [24], [27], [34], [38]. Many students reported shifts in their attitudes and empathy toward patient care [20], [21]. Complementing these qualitative accounts, studies with quantitative results found statistically significant improvements in attitudes toward patients and higher empathy levels following the simulation experiences [28], [36], [41]. Students described increased patience, a stronger commitment to preserving dignity and autonomy, and a renewed focus on listening and being present [29], [33]. Some students even characterized the experience as life-changing, noting that they would now approach patients with greater humility, attentiveness, and empathy [20], [35]. Additionally, two studies explored how the learning from the simulations translated into clinical practice. One study used a follow-up questionnaire to assess changes in students’ empathy and altruism levels [30], while the other study directly observed students during their clinical experiences to evaluate the application of skills and empathy gained from the simulation [20].

#### 3.4.3. Theme 3: Ethical and pedagogical considerations 

Educators emphasized the importance of creating a supportive and safe learning environment where students can engage in simulations without fear of harm or judgment [29], [35]. Several studies highlighted the pedagogical value of low-cost, immersive tools when paired with clear instructional goals, reflection, and thoughtful integration into broader curricula [23], [36], [40]. Virtual reality (VR) was also noted as an effective, relatively low-cost tool that can enhance immersion and perspective-taking in simulation-based learning [28], [36], [41]. To ensure realism and relevance, researchers recommended grounding simulations in lived experience narratives, involving patients in development [27], [29], or using movies and case studies [28]. Preparation and debriefing were repeatedly stressed as essential components. A well-structured pre-briefing phase helped students better embody the patient perspective [29], [40]. One study reported that students struggled to fully engage as patients, especially when lacking clinical experience [30]. Accordingly, several authors recommended targeting more experienced students or integrating simulation with other activities, such as drama, interviews, or discussion-based courses, to deepen engagement and increase transferability of learning [21], [30]. While the studies reviewed were generally encouraging about the utility of these simulations, researchers also raised important limitations. Simulations, by design, offer only brief, artificial glimpses into what are often lifelong and complex experiences. They may not fully replicate the chronic, cumulative realities of living with disability, illness, or aging [35]. Additionally, poorly designed or insufficiently contextualized simulations carry risks of oversimplification, reinforcing stereotypes, or inadvertently increasing anxiety [22], [35].

## 4. Discussion

In reflecting on the use of simulated patient perspective exercises within undergraduate nursing education, this scoping review opens a broader discussion around both the potential benefits and significant challenges associated with these educational tools. As the first comprehensive attempt to map this landscape within nursing education, the review highlights the potential of important themes, yet it also invites further dialogue regarding the impact and implementation of these exercises. Across the included studies, three themes emerged:

1. students’ immediate responses during the exercises,
2. the perceived impact after participation, and
3. ethical and pedagogical considerations around implementation.

These findings align with prior research showing that simulations that place learners in the role of a patient may be particularly effective in fostering empathy compared to other teaching methods [43]. For undergraduate nursing students, who will inevitably care for patients with complex health conditions, the ability to take another’s perspective is described as a foundational tool of nursing practice, one that requires not only clinical reasoning but also attention to the subjective dimensions of patient experience [5], [44]. From this perspective, patient perspective simulations may represent a valuable strategy for helping students bridge the gap between theoretical knowledge and the lived realities of patients and their families.

While the findings suggest that these exercises can deepen students’ understanding of patient experiences, the review also underscores several practical, ethical, and methodological challenges that should be considered to enhance their educational value. The immersive nature of these simulations appears powerful in shaping students’ perspectives, often described as “eye-opening” and more effective than traditional classroom instruction [23], [24], [33]. These experiences frequently provoked strong emotional and even physical responses: students reported feelings of frustration, anxiety, disorientation, vulnerability, and sadness [23], [31], [37], [42]. Although initially distressing, many students later described these responses as meaningful and transformative, noting that the intensity of the experience made them more aware of what it might feel like to live with a disability, dementia, or mental illness [20], [22], [27]. Several studies highlighted that this discomfort, when it is properly supported by debriefing, helped students develop more empathy, patience, and a stronger focus on individualized care. In this way, emotional discomfort was not seen as a problem, but as an important part of the learning process that encouraged reflection and changes in attitude. This is in line with earlier research showing that challenging experiences can support personal and professional growth [13]. However, while students frequently reported short-term insight and emotional impact, only two studies explored how these shifts translated into sustained behavioral change or clinical practice improvements [20], [30]. In one study, follow-up questionnaires indicated no measurable changes in attitudes or behaviors, suggesting limited durability of the simulation effects [30]. In contrast, observational data from the other study suggested that students applied some of the skills and perspectives gained from the simulations during clinical practice, although the timeframe and duration of these observations were not clearly reported, making it difficult to assess the persistence of these effects [20]. The importance of structured prebriefing and debriefing was consistently emphasized across studies as a key success factor in the design of patient perspective simulations [20], [23], [31], [36]. These stages, which are endorsed by best-practice standards from the International Nursing Association for Clinical Simulation and Learning (INACSL), serve not only to prepare students cognitively, but also to ensure emotional readiness [45]. Prebriefing strategies included theoretical framing, scenario explanations, and the use of lived-experience narratives, all aimed at helping students engage more meaningfully with the perspective they were about to embody [23], [40]. Emotional safety was often explicitly addressed: students were informed of their right to withdraw, and facilitators were trained to support those who experienced distress during or after the simulation [23], [40]. Debriefing served as an important pedagogical and ethical component, offering students a space for critical reflection and emotional processing [46]. These sessions in the form of group discussions, written reflections, or one-on-one facilitation helped students articulate the complex emotions elicited by the experience and translate them into empathetic understanding [20], [27], [41]. Importantly, debriefing also functioned as a safeguard for student well-being. As Schmidt et al. argue, structured debriefing is crucial for recognizing and addressing adverse reactions or internal conflicts that may arise, particularly when simulations involve sensitive topics like disability or mental illness [12]. If left unprocessed, these responses risk reinforcing stereotypes or impeding learning. Incorporating narratives from people with lived experience and engaging them in simulation design can enhance both the authenticity and ethical grounding of the exercise [2], [9].

A key concern in the literature is the authenticity and ethical framing of simulations, particularly when they involve disabilities or mental health conditions. Scholars have cautioned that such exercises can oversimplify lived experiences and inadvertently perpetuate negative assumptions [7], [8], [9]. Some even argue that perspective-taking may reinforce rather than dismantle stereotypes, especially when the simulated group is already socially marginalized [47]. For instance, Skorinko and Sinclair [48] highlight that when learners rely on pre-existing stereotypes to imagine another’s experience, simulations risk entrenching biased views. Similarly, Silverman [8] critiques disability simulations for emphasizing the initial “shock” of becoming disabled, while neglecting the long-term adaptation, resilience, and autonomy many individuals develop, thereby framing disability as tragic and disempowering. Nario-Redmond et al. [49] further contend that these activities may fail to improve attitudes toward disabled individuals and can even undermine integration efforts. Interestingly, such concerns were rarely addressed in the studies included in this review, pointing to a disconnect between theoretical critiques and the way simulations are currently evaluated in nursing education. This gap underscores the need for more explicit ethical framing and for the active involvement of people with lived experience in designing and delivering these exercises, to ensure that simulations not only foster empathy but also avoid misrepresentation and stigmatization.

### 4.1. Limitations and strengths

The following section outlines the main methodological limitations of this scoping review before highlighting its strengths. As with all scoping reviews, the aim of this study was to map the breadth of existing research rather than evaluate the effectiveness or quality of interventions. Consequently, while thematic analysis enabled the identification of overarching patterns across studies, a more focused qualitative synthesis could offer nuanced interpretations.The studies reviewed varied widely in methodology, participant demographics, intervention design, and educational settings. Although this heterogeneity is expected in a scoping review, it poses challenges for synthesizing findings and consistently identifying patterns. The exclusion of non-English and non-German publications introduces a potential language bias, as relevant studies published in other languages may have been missed. Moreover, the exclusion of grey literature might also have introduced publication bias, since studies reporting positive results are more likely to appear in peer-reviewed journals. Furthermore, study selection was conducted primarily by the first reviewer, without duplicate independent screening, which may increase the risk of selection bias despite efforts to critically reassess decisions with the research team.

Despite these limitations, the methodological design of this scoping review also presents several strengths. The use of a transparent, systematically documented search strategy across multiple databases, adherence to PRISMA-ScR reporting guidelines, and structured data charting enhance the rigor and reproducibility of the review. Collaboration among the research team during data extraction and thematic synthesis further contributed to analytical consistency and reflexivity. Collectively, these elements strengthen the validity, transparency, and utility of the review findings.

In addition to these methodological strengths, certain characteristics of the included studies themselves can also be viewed as strengths of the existing evidence base. The diversity of the included studies illustrates the creativity with which nursing educators have approached patient-perspective simulations and the adaptability of such exercises across different contexts. The inclusion of various types of simulation, ranging from role-play to immersive technology-based interventions, provides a rich and multifaceted picture of how empathy and perspective-taking can be fostered in undergraduate nursing education.

## 5. Conclusion

In conclusion, this scoping review suggests that patient perspective simulations, despite some implementation challenges, are generally perceived by students as valuable tools for enhancing empathy, deepening understanding of patient needs, and fostering a more patient-centered approach in nursing education. The emotional reactions experienced, such as anxiety and confusion, are often seen as valuable entry points for deeper learning. However, to ensure psychological safety and educational value, simulations must be carefully designed with structured prebriefing and debriefing. 

Future research should focus on optimizing the design and implementation of patient perspective exercises, particularly to understand experiences of students who respond negatively or disengage. Longitudinal studies are essential for assessing whether short-term insights translate into lasting changes in attitudes and clinical practices, especially during placements or early career stages. Expanding these exercises across diverse cultural and institutional settings could provide insights into contextual factors influencing outcomes.

## Authors’ ORCIDs

- Anna Christine Steinacker: [0009-0000-4466-1407]
- Michael Klingenberg: [0000-0003-1324-5210]
- Stefan Böser: [0000-0002-3095-4396]

## Competing interests

The authors declare that they have no competing interests. 

## Supplementary Material

PubMed search query November 2024

Summary of simulation types and study designs

## Figures and Tables

**Table 1:**
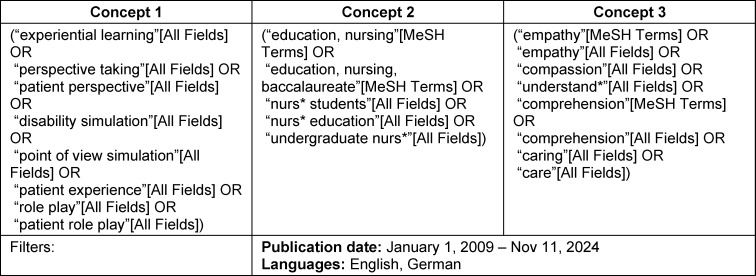
PubMed search terms
