# Supplementary material for: Implementation and impact of simulated patient perspective exercises in undergraduate nursing education: A scoping review
Source: GMS J Med Educ. 2026 Apr 15;43(4):Doc53. doi: 10.3205/zma001847 (PMC13124500; doi:10.3205/zma001847)
Supplement: PubMed search query November 2024 [file JME-43-53-s-001.pdf]

Attachment 1: PubMed search query November 2024

#	**Query**	Results
**#1**	"experiential learning"[All Fields] OR "perspective taking"[All Fields] OR "patient perspective"[All Fields] OR "disability simulation"[All Fields] OR "point of view simulation"[All Fields] OR "patient experience" OR "Role Play" OR "Patient role play"	27,837
**#2**	"education, nursing"[MeSH Terms] OR "education, nursing, baccalaureate"[MeSH Terms] OR "nurs* students"[All Fields] OR "nurs* education"[All Fields] OR "undergraduate nurs*"[All Fields]	131,191
**#3**	"empathy"[MeSH Terms] OR "empathy"[All Fields] OR "empathy"[MeSH Terms] OR "empathy"[All Fields] OR "compassion"[All Fields] OR "comprehension"[MeSH Terms] OR "comprehension"[All Fields] OR "understand"[All Fields] OR "understanding"[All Fields] OR "understands"[All Fields] OR "understandability"[All Fields] OR "understandable"[All Fields] OR "understandably"[All Fields] OR "understandings"[All Fields] OR "care s"[All Fields] OR "cared"[All Fields] OR "carefulness"[All Fields] OR "cares"[All Fields] OR "empathy"[MeSH Terms] OR "empathy"[All Fields] OR "caring"[All Fields]	2,067,991
**#4**	#1 AND #2 AND #3	374
**#5**	#1 AND #2 AND #3<br>Filters: 2009-2024; English	288

Attachment 1 to Steinacker AC, Klingenberg M, Böser S. *Implementation and impact of simulated patient perspective exercises in undergraduate nursing education: A scoping review.* GMS J Med Educ. 2026;43(4):Doc53. DOI: 10.3205/zma001847
