# Supplementary material for: Implementation and impact of simulated patient perspective exercises in undergraduate nursing education: A scoping review
Source: GMS J Med Educ. 2026 Apr 15;43(4):Doc53. doi: 10.3205/zma001847 (PMC13124500; doi:10.3205/zma001847)
Supplement: Summary of simulation types and study designs [file JME-43-53-s-002.pdf]

Attachment 2: Summary of simulation types and study designs

	Author, year, country	Titel of article	Type of study	Implementation	Emotions	Evaluation
Hearing Voices that are distressing' - Simulation Package	Hamilton Wilson et al, 2009, United States	A Narrative Study of the Experiences of Student Nurses Who Have Participated in the Hearing Voices that are Distressing Simulation	Qualitative study<br>**Participants**: 27 nursing Students<br>**Data collection:** Students responded in writing to three open ended prompts<br>**Data analysis**: Thematic analysis	The "Hearing Voices" simulation was conducted in small groups within a classroom, beginning with orientation and a preparatory video, followed by a 45-minute simulation using CD players with recordings of distressing voices. Participants engaged in various activities (e.g., crafts, card games, reading).	Students reported a profound impact, with enhanced empathy and greater respect for individuals experiencing distressing voices. Many expressed relief at being able to stop the recording and reflected on the emotional stress of auditory hallucinations.	The simulation was well received by all nursing students, who valued the experiential insight it provided into living with auditory hallucinations.
	Chaffin & Adams, 2013, United States	Creating Empathy Through Use of a Hearing Voices Simulation	Mixed-methods<br>**Participants:** 67 nursing students<br>**Data collection:** Survey on Likert-type scale measures of students' self-rated empathy pre- and post simulation and written reflections after the simulation experience<br>**Data analysis:**<br>A *t*-test analysis on pre–post empathy ratings; reflections were analysed by content theme analysis	Students completed the "Hearing Voices" simulation using the curriculum's instructor guide, which involved listening to recorded voices via portable CD players while performing six sequential tasks. Each task was set up at individual classroom stations with supplies and instructions prepared in advance. Students began at Station 1 and progressed through all stations in order, attempting each task while wearing headphones.	Debriefing revealed strong emotional reactions, including confusion, frustration, anger, worthlessness, disappointment, tiredness, irritation, impatience, and empathy. Many students reported being deeply moved and stated they would approach psychiatric patients with greater understanding in the future.	Analysis conducted before and after the simulation showed notable increases in students' empathy levels. Content analysis revealed that students experienced a transformative effect, reporting a deeper understanding of psychiatric patients and a greater commitment to practicing patience in their interactions. Notably, these attitudinal shifts translated into observable behavioral changes in subsequent clinical practice.

	Author, year, country	Titel of article	Type of study	Implementation	Emotions	Evaluation
	Orr et al., 2013, Australia	The distress of voice-hearing: The use of simulation for awareness, understanding and communication skill development in undergraduate nursing education	Qualitative design **Participants:** 76 nursing students **Data collection:** Students filled out written evaluation forms **Data analysis:** Thematic analysis of answers	Students received mp3 players with 45-minute voice-hearing recordings and instructions for safe use. Each tutorial group was split into two sub-groups of 10, completing tasks both inside (reading, group discussion) and outside the classroom (library, cafeteria, email, phone calls). Participants could discreetly stop playback if distressed.	Participants most frequently reported irritability/disturbance (52.6%), fear/distress (43.4%), and self-consciousness (13.2%). Behavioral effects included reduced concentration (42.1%), desire to respond to voices (35.5%), and altered social interactions (26.3%).	The workshop enhanced understanding of voice-hearing, increased awareness of its impact on daily functioning, and improved insight into communication strategies for engaging with voice-hearers. Participants reported valuable empathy gains and an ability to identify effective, respectful communication approaches.
	Mawson, 2014, Australia	Use of media technology to enhance the learning of student nurses in regards to auditory hallucinations	Mixed-methods design **Participant: 60** nursing students **Data collection:** Pre-/Posttest with open ended-questions **Data analysis:** paired sample t-test. The open-ended responses were analysed to identify frequently-occurring responses	Student nurses worked in pairs during a classroom role-play using iPods. One student listened to a 5-minute simulation of auditory hallucinations while being interviewed by the other student. Roles were then swapped.	Students reported feelings of frustration, irritability, discomfort, and being disturbed by the experience.	Nine of eleven measured items showed statistically significant improvements This media-based simulation increased students' knowledge and appreciation of patients' lived experiences, reinforcing therapeutic communication

	Author, year, country	Titel of article	Type of study	Implementation	Emotions	Evaluation
	Fossen & Stoeckel 2016, United States	Nursing Students' Perceptions of a Hearing Voices Simulation and Role-Play: Preparation for Mental Health Clinical Practice	Qualitative design **Participants**: 40 nursing students **Data collection:** Survey with open-ended questions **Data analysis:** qualitative interpretive phenomenological method	The 5-hour intervention combined the *Hearing Voices That Are Distressing* simulation with a role-play exercise. Activities included an orientation, the simulation across five task stations (puzzles, math problems, address form, journal article summary, door counting), a role-play of a nurse–patient scenario, a debriefing, and surveys. In the role-play, participants alternated between acting as a nurse and as a patient.	Before the intervention, students reported fear, anxiety, uneasiness, and uncertainty about communicating with patients who hear voices, often describing such interactions as "scary" or "confusing." The experience shifted perceptions, fostering empathy and understanding of agitation, irritability, and fear in those with auditory hallucinations.	Students developed more patient, open-minded, understanding, and nonjudgmental approaches to communication. The simulation deepened understanding of the challenges faced by individuals with auditory hallucinations, while the role-play offered practice in applying these insights. Together, these methods better prepared students for clinical work with patients experiencing voice-hearing.
	Langham et al. 2016, United States	Transforming future nurses through simulation in mental health nursing	Qualitative design **Participants**: 51 nursing students. **Data collection**: Pre- and post-simulation written narratives. **Data analysis**: Narrative content analysis.	Students tried the "Hearing Voices" simulation using MP3 players with recordings of distressing voices. Students progressed through six activity stations in any order: (1) recall exercise, (2) word search, (3) reading/comprehension, (4) creative writing, (5) math/money counting, (6) matchstick puzzle	Students initially expressed negative or stigmatizing attitudes (e.g., "crazy", "faking voices") but reported increased understanding and empathy after the simulation. Many described anxiety, frustration, and disorientation during the tasks, alongside a growing awareness of the challenges faced by individuals experiencing auditory hallucinations	The simulation was found to positively influence nursing students' attitudes, empathy, and understanding. The authors concluded that the exercise provides further support for "hearing voices" simulations as an effective teaching strategy in mental health nursing.

	Author, year, country	Titel of article	Type of study	Implementation	Emotions	Evaluation
Simulated Ostomate	Díaz et al. 2015, United States	Creating Caring and Empathic Nurses: A Simulated Ostomate	Qualitative design<br>**Participants:** 69 nursing students<br>**Data collection:** Students wrote a written reflection<br>**Data analysis:** Descriptive phenomenology by Colaizzi (1978)	Students wore an ostomy appliance filled with simulated content for up to 48 hours as part of the assignment. Although some students did not complete the full duration due to emotional or physical challenges, all engaged with the simulation and reflected on their experiences.	Participants expressed a wide range of emotions including anger and sadness. Five key themes emerged related to reactions to wearing the ostomy, impacts on body image and relationships, and growing empathy.	The simulation was described as valuable and insightful, offering participants a deeper understanding of living with an ostomy. Many participants reported life-changing experiences, suggesting that such simulations provide important benefits for future nursing practice
	Maruca et al., 2015, United States	Enhancing Empathy in Undergraduate Nursing Students: An Experiential Ostomate Simulation	Quantitative design<br>**Participant:** 69 nursing students<br>**Data collection**: Multi dimensional empathy scale<br>**Data analysis:** Quantitative content analysis by Krippenhoff	Students wore a stoma system with simulated stool over a weekend (Friday to Sunday). On Sunday, they submitted a “proof photo” documenting the wearing of the appliance.	Students expressed worry about having the disease or ostomy. The experience was described as an eye-opener, leading to a better understanding of what patients go through.	85% of students found the simulation a valuable learning tool that enhanced their understanding of patient experiences and improved their empathy. This study supports the use of such simulations to foster empathy in nursing students.
	Hood et al. 2018 United States	Stepping into their shoes: The ostomate experience	Qualitative design<br>**Participants**: 30 nursing students<br>**Data collection:** Reflection Papers<br>**Data analysis**: Thematic analysis	**Implementation:** Nursing students (n=30) applied ostomy systems to one another using case cards. The stoma bags were filled with simulated stool and worn for 24 hours.	Students reported feelings of worry and embarrassment, but also excitement at experiencing the patient perspective. Several expressed relief and gratitude that the experience was only temporary.	The simulation was considered an effective strategy for gaining insight into the patient’s point of view. While some students were initially hesitant, most later expressed gratitude for participating in the exercise.

	Author, year, country	Titel of article	Type of study	Implementation	Emotions	Evaluation
	Swenty & Doerner 2023, United States	Nursing Students' Perceptions Following an Ostomy Experiential Activity: A Qualitative Study	Qualitative design **Participants**: 145 nursing students **Data collection**: written assignment after wearing ostomy bag **Data analysis**: thematic analysis	Students wore a stoma system filled with 30–50 ml of water during a four-hour skills lab session, including breaks. Nursing skills related to ostomy care were practiced.	Students reported a wide array of emotional responses, embarrassment, worry to moments of forgetting the appliance was present. Simulations was exe-opening and helped to identify obstacles in daily tasks by patients.	Overall, students felt the experience increased their knowledge and empathy, enhancing their ability to care for patients with ostomies. The study identified themes around emotional responses, understanding patients' experiences, and positive learning outcomes, indicating effective emotional learning transfer to patient care.
**“ Day in the Life of a Patient”**	Levett-Jones et al. 2017 Australia	Measuring the impact of a 'point of view' disability simulation on nursing students' empathy using the Comprehensive State Empathy Scale	Quantitative design **Participants:** 360 nursing students **Data collection**: A two-group Pre/Post test design using the comprehensive state empathy scale. **Data analysis**: t- test	Paired Students were assigned roles of either a person with acquired brain injury (ABI) or a rehabilitation nurse. The 'patient' wore a hemiparesis suit simulating dysphasia, hemianopia, and hemiparesis and performed tasks such as dressing and walking.	Individual emotional responses were not reported	The study showed that immersive point-of-view simulations can effectively enhance learners' empathy towards vulnerable patient populations, highlighting the potential for such methods to positively impact clinical practice.
	Ter Beest et al. 2018 Netherlands	Nursing student as patient: experiential learning in a hospital simulation to improve empathy of nursing students	Qualitative design **Participants**: 96 nursing students **Data collection:** Open ended questions **Data analysis**: Coding by Corbin & Strauss	Students simulated hospital patient experiences over four hours in a nursing education institute's mock hospital rooms. They were fitted with patient attributes (e.g., fake stoma, bandages), and lived as patients alongside peers. Nursing students acted as nurse.	Students experienced shock and eye-opening realizations about the passivity, boredom, and emotional isolation of being a patient. Reported feelings included loneliness, shame, feeling forgotten, and vulnerability.	The simulation deepened students' empathy by immersing them in the patient's world, highlighting the challenges patients face and emphasizing the importance of empathy in nursing care.

	Author, year, country	Titel of article	Type of study	Implementation	Emotions	Evaluation
	Levett-Jones et al. 2018, Australia	Exploring Nursing Students' Perspectives of a Novel Point-of-View Disability Simulation	Mixed- methods<br>**Participants**: 384 nursing students<br>**Data collection**: Satisfaction with Disability Simulation Experience Scale (SDSES) questionnaire and opened ended question<br>**Data analysis**: Descriptive and inferential statistics. Thematic analysis of open-ended questions	Students were randomly assigned to either the role of a person with acquired brain injury (ABI) or a rehabilitation nurse. The ABI role involved wearing a hemiparesis suit with leg brace, weights, sling, ear plugs, walking stick, and hemianopia glasses to simulate physical impairments.	Participants valued the novel, eye-opening experience, gaining deeper understanding and empathy toward those living with ABI. Some wished for the chance to experience both roles. The simulation fostered feelings of "standing in someone else's shoes," respect, and curiosity about living with physical limitations.	High satisfaction and positive feedback highlight the simulation's effectiveness in fostering empathy and new insights. Participants reported it would enhance their future nursing practice and support investing in experiential learning to improve empathic patient car
	Basit et al. 2023 Turkey	The effect of drama-supported, patient role-play experience on empathy and altruism levels in nursing students: A randomized controlled study	Quantitative design<br>**Participants**: 52 nursing students<br>**Data collection:** Pre/Post-test design. Data was collected using a descriptive characteristics form, the altruism scale and the Jefferson scale of Empathy for Nursing Students (JSENS).<br>**Data analysis**: CHI square, ANOVA	Participants played the role of a bed-bound patient with case details provided shortly before the session. Professional nurses cared for up to seven patient-role students in a simulated skills lab with three patient rooms. Each role-play lasted 4 hours, and the intervention ran over two days	Individual emotional responses were not reported	The intervention group showed a significant increase in altruism compared to the control group, but no differences were found in empathy scores, including perspective-taking. Although the patient role-play improved empathy and altruism initially, the effect did not persist at the three-month follow-up.

	Author, year, country	Titel of article	Type of study	Implementation	Emotions	Evaluation
	Witherspoon et al., 2023, United States	The impact of a role-play patient simulation on nursing students as measured by the comprehensive state empathy scale	Quantitative design<br>**Participants**: 83 nursing students<br>**Data collection**: Pre/Post test design using the Comprehensive State Empathy Scale (CSES) questionnaire.<br>**Data analysis**: Paired samples t-test	Students were paired and alternately took on the role of an older patient with physical limitations or a nurse. Students wore simulation equipment (scuffed eyewear, ear plugs, gloves, balance pad) to mimic sensory and mobility impairments. “Patients” completed tasks such as reading an insurance card, managing medications, and paying while standing on a balance pad.	Individual emotional responses were not reported	Participants engaged in the role-play simulation, which led to a statistically significant increase in CSES scores. These results support the effectiveness of role-play simulations in enhancing empathy in nursing students.
**Sensory Loss**	Nasrabadi et al., 2021, Iran	Exploring nursing students' experiences of blindness simulation: A phenomenological study	Qualitative design<br>**Participants**: 8 nursing students<br>**Data collection**: Semi-structured interviews were conducted<br>**Data analysis**: Descriptive phenomenology by Colaizzi	Participants were blindfolded for at least 3 hours, preventing them from seeing or perceiving light, and given canes to avoid injury. During this time, they continued their usual activities at school, such as attending classes, navigating hallways, eating, socializing, praying, and more.	Students reported feelings of abandonment, fear, and emotional isolation, describing the experience as living in ambiguous darkness and an unpredictable environment. They also noted increased reliance on other senses, a sense of self-alienation, dependence, and reduced self-confidence.	The simulation shifted students’ attitudes, fostering a deeper understanding of the needs of blind individuals. Participants felt better equipped to provide care and support after the exercise. The study highlights how such simulations can enhance empathy and inform community and policy interventions to better support the blind.

	Author, year, country	Titel of article	Type of study	Implementation	Emotions	Evaluation
	Hannans et at., 2021, United States	See it, hear it, feel it: embodying a patient experience through immersive virtual reality	Quantitative design **Participants:** 165 nursing students **Data collection:** Pre /Post test before and after virtual experience concerning embodiment **Data analysis**: comparison of Pre-/ Post-survey means	Two immersive VR experiences were piloted with junior and senior nursing students. In *We Are Alfred*, students embodied a 76-year-old man with hearing loss and macular degeneration, In *The Beatriz Lab: A Journey Through Alzheimer's*, students embodied a middle-aged woman progressing through early, middle, and late stages of Alzheimer's Disease.	Individual emotional responses were not reported	Immersive VR effectively integrated into the nursing curriculum, significantly improving students' knowledge, confidence, and empathy across three cohorts. Participants gained perspective on living with chronic conditions and disease progression, enhancing their ability to relate to and care for patients. Results suggest VR's potential as a cost-effective, experiential teaching method that addresses both cognitive and affective learning outcomes.
**Virtual Dementia Tour experience**	Deprey & Kobiske, 2023, United States	Dementia Simulation Impact on Empathy of Nursing and Physical Therapy Students: A Quantitative Study	Quantitative design **Participants:** 248 nursing and PT students **Data collection:** Pre/Post test design using the interpersonal reactivity index, perspective taking (PT), and empathic concerns (EC) subscales **Data Analysis**: Non-parametric statistics	Participants donned simulation equipment designed to replicate the sensory and cognitive changes experienced by individuals with dementia This included vision-limiting goggles, headphones with background noise, tactile-impairing gloves, and shoe inserts. The VDT room, staged as a small one-bedroom apartment, contained five simple tasks for participants to complete within eight minutes.	During the debrief, participants recognized parallels between their own reactions in the simulation and common behaviors of persons with dementia, such as wandering, hoarding, agitation, and difficulty concentrating.	Pre- and post-survey results using the Interpersonal Reactivity Index (IRI) demonstrated a statistically significant increase in empathy scores, specifically in perspective-taking and empathic concern, after participation. The VDT experience enhanced understanding of the daily challenges faced by individuals with dementia and was found to be an effective method for increasing empathy among nursing and DPT students.

	Author, year, country	Titel of article	Type of study	Implementation	Emotions	Evaluation
	Morales et al. 2024 United States	The effect of combining an e-learning module with s Virtual Dementia Tour® on knowledge and attitudes toward person-centered dementia care in prelicensure nursing education	Quantitative design **Participants:** 121 nursing students **Data collection:** cross-over and Pre/Post test design **Data analysis**: SPSS	The Virtual Dementia Tour (VDT) kit provided students with an eight-minute immersive experience of altered sensory perceptions, simulating life with dementia. Students wore glasses to cloud and narrow their vision as overhead lighting was dimmed, headphones emitting confusing background noises, thick gloves, and shoe inserts to impair touch and mobility, they were tasked with completing five simple activities.	During debriefing, students described feeling overwhelmed, frustrated, and disoriented. They reported heightened empathy for individuals living with dementia, noting the difficulty of hearing and following instructions in the simulated environment. Many participants began "looking for something to do," only to realize these actions could be perceived by others as wandering, rummaging, or hoarding—mirroring common misinterpretations of dementia-related behaviors.	The study found that direct experiential learning through VDT was a stronger predictor of improved attitudes toward dementia care than prior education alone. Students who completed an e-learning module before the simulation scored higher in post-activity attitudes compared to those who completed it afterward. These results suggest that sequencing educational tools strategically can maximize empathy and effectiveness in dementia care.
**Age Suit**	Bouwmeester Stjernetun et al., 2024, Sweden	Effects of an age suit simulation on nursing students' perspectives on providing care to older persons - an education intervention study	Quantitative design **Participants**: 471 nursing students **Data collection:** Quasi-experimental Pre/Post test design **Data analysis:** Mann-Whitney U analysis	Each student was assigned a unique *persona* representing specific age-related health problems such as vision and hearing impairments, musculoskeletal dysfunctions, neurological disorders, chronic pain, or chronic obstructive lung disease. Students wore an age suit that mimicked these conditions. Students completed five everyday living tasks over approximately 50 minutes. Activities included setting a dinner table, brushing teeth, resting in bed.	Individual emotional responses were not reported	The intervention positively influenced nursing students' attitudes toward working with older adults. While the total mean score increased post-intervention (not statistically significant), improvements were seen in specific items. The intervention group expressed greater interest in caring for older persons as a career choice compared to the control group. Frustration scores related to working with older persons were also higher post-intervention in the intervention group.

	Author, year, country	Titel of article	Type of study	Implementation	Emotions	Evaluation
	Bouwmeester Stjernetun et al., 2024, Sweden	”It´s like walking in a bubble”, nursing students´ perspectives on age suit simulation in a home environment – group interviews from reflection seminars	Qualitative design<br>**Participants**: 39 nursing students<br>**Data collection**: Semi-structured interviews<br>**Data analysis**: Reflexive thematic analysis	The Age Suit was used to simulate ageing and age-related health conditions. The suit included straps, weights, eyeglasses, gloves, and headphones to mimic hemiparesis, impaired vision, eye diseases, tremor, tinnitus, restricted movement, and unstable gait. Each nursing student was assigned one of eight *personas* with specific health problems where students performed ordinary daily tasks such as getting in and out of bed, brushing teeth, combing hair, writing a simple message, cleaning, and setting the table.	Students described the experience as “like walking in a bubble,” with subthemes of “moving in a heavy body drains energy,” “losing senses is frustrating and frightening,” and “feeling lonely and disconnected.” Many considered it “an eye opener,” particularly in realizing the difficulty of coping with cognitive loss.	Age suit simulation provided an immersive, eye-opening experience, enhancing students' awareness of the physical and cognitive challenges older adults face, including vulnerability and the need for patience and presence in care. Students reported feeling motivated to apply these insights in clinical practice, recognizing age suit simulation as a valuable addition to geriatric education
**Self-Testing**	Chiou et al. 2019 Taiwan	Experiential learning of HIV self-test among student nurses: A qualitative Study	Qualitative design<br>**Participants**: 30 nursing students<br>**Data collection**: Students were interviewed during the waiting period<br>**Data analysis**: Line-by-line content analysis	A Rapid HIV-1/2 Antibody Test was used as the self-testing tool. Each participant received 30 minutes of pre-test counselling covering HIV transmission, confidentiality, and the implications of results. Participants took the testing kit home and conducted the self-test following official procedures.	During the self-testing process, participants experienced emotional fluctuations, isolation from the outside world, and questioned the test’s accuracy and safety. Some preferred to view results immediately, while others delayed. In the post-test period, participants reported uncertainty about the result, reflected on the impact and needs related to self-testing, and in some cases felt a sense of loss.	Participants reflected on the impact and needs related to self-testing. Some reported delaying result viewing to cope with anxiety, while others viewed results immediately. The study highlighted how self-testing prompted considerations of emotional support, the importance of clear instructions, and the need for follow-up services to address uncertainty and psychological responses.
